# Prognostic value of HRCT-based risk stratification for acute/subacute progression in polymyositis/dermatomyositis-associated interstitial lung disease

**DOI:** 10.3389/fimmu.2026.1748191

**Published:** 2026-01-28

**Authors:** Siyu Jiang, Kaixiang Su, Caifeng Pang, Yuqing Tang, Yujie Xiang, Ju Han, Hongji Pu, Yonglong He, Rui Li

**Affiliations:** 1 Department of Radiology, Affiliated Hospital of North Sichuan Medical College, Nanchong, China; 2 Department of Radiology, Affiliated Stomatological Hospital of Qujing Health Medical College, Qujing, China; 3 Department of Rheumatology and Immunology of the Affiliated Hospital of North Sichuan Medical College, Nanchong, China

**Keywords:** acute or subacute progression, computed tomography, dermatomyositis, interstitial lung disease, polymyositis, prognosis, risk stratification

## Abstract

**Objectives:**

Aiming to evaluate the predictive value of high-resolution computed tomography (HRCT) features for identifying acute/subacute progression in patients with polymyositis/dermatomyositis (PM/DM)-associated interstitial lung disease (ILD), and to develop a risk stratification algorithm based on clinico-radiologic parameters.

**Methods:**

This retrospective cohort study included 282 patients with PM/DM who underwent HRCT from January 2020 to December 2024. Baseline clinical data and HRCT imaging characteristics were systematically collected. Over time, 140 patients with PM/DM-ILD were followed. HRCT scores and imaging patterns were assessed, and cases of acute/subacute ILD progression were documented during the follow-up period. Penalized Cox regression (LASSO) was conducted to identify independent risk factors associated with disease progression and to develop a risk stratification method. The concordance index (C-index), net reclassification improvement (NRI), integrated discrimination improvement (IDI) and decision curve analysis (DCA) were used to evaluate the discriminative ability of this stratification. Algorithm performance was assessed using calibration plots to evaluate agreement between predicted and observed risks.

**Results:**

During a median follow-up duration of 5.69 months (IQR, 1.77–5.91 months), 56 (40.0%) patients experienced acute/subacute ILD progression. The HRCT score was considered an independent predictor of acute/subacute progression in patients with PM/DM-ILD. A newly developed risk stratification scheme, according to thresholds of HRCT score, imaging classification (organizing pneumonia [OP] vs. non-OP patterns), and anti-MDA5 antibody status, demonstrated good predictive ability for identifying patients at risk of progression. In combination with clinical parameters, the integrated predictive algorithm significantly outperformed traditional clinical risk algorithm, with significant enhancements in C-index (to 0.764). The incremental predicted value was demonstrated by improved NRI (0.470), IDI (0.218), and DCA metrics.

**Conclusion:**

A high HRCT score is an independent predictor of acute/subacute progression in patients with PM/DM-ILD. Incorporating clinical parameters into the imaging-based algorithm significantly improves its predictive accuracy for progressive disease.

## 1 Introduction

Polymyositis (PM) and dermatomyositis (DM) are idiopathic inflammatory myopathies categorized by chronic proximal muscle inflammation, with DM presenting cutaneous manifestations (1). Besides muscular and dermatologic involvement, the respiratory system is frequently affected, with interstitial lung disease (ILD) representing one of the most prevalent and life-threatening complications (2). The global prevalence of PM/DM-associated ILD (PM/DM-ILD) is approximately 41.0%, with a notably higher prevalence among Asian populations compared with North American and European cohorts (3). PM/DM-ILD is histopathological heterogeneous, predominantly presenting with patterns, including nonspecific interstitial pneumonia (NSIP), organizing pneumonia (OP), NSIP plus OP, usual interstitial pneumonia (UIP), and diffuse alveolar damage (DAD) (4). Importantly, a subset of patients develops rapidly progressive ILD (RP-ILD) or subacute progressive ILD, characterized by the abrupt onset of worsening hypoxemia, extensive bilateral pulmonary infiltrates on imaging, and progression to respiratory failure over a short time. These patients frequently require intensive care and mechanical ventilation. The condition is associated with a high mortality rate (2, 5).

Recent studies have revealed that compared with conventional treatments, early initiation of combination immunosuppressive therapy significantly improves survival outcomes in patients with PM/DM-ILD (6). Accordingly, timely diagnosis and precise risk stratification are crucial for optimizing clinical decision-making and improving prognosis in this high-risk patient population.

Several clinical and serological markers have been identified for predicting ILD occurrence and progression in patients with PM/DM. Among them, anti-melanoma differentiation-associated gene 5 (anti-MDA5) antibodies have been a key prognostic biomarker, strongly associated with disease progression and increased mortality (7). Clinical factors, including serum C-reactive protein (CRP), ferritin, and Krebs von den Lungen-6 levels, have been implicated in developing acute or subacute ILD progression in patients with PM/DM (8). However, most existing studies have primarily focused on individuals who are anti-MDA5-positive (9), whereas predicting disease progression in patients who are anti-MDA5-negative remains a significant clinical challenge. Therefore, developing comprehensive risk stratification algorithm that incorporate both serological and nonserological indicators is urgently needed to improve prognostic accuracy across the broader PM/DM-ILD population.

High-resolution computed tomography (HRCT) is the gold standard for diagnosing ILD, providing a precise assessment of the extent and pattern of pulmonary involvement (10). HRCT-based assessment commonly employs qualitative and semi-quantitative scoring systems and imaging pattern and lesion distribution evaluation, contributing to risk stratification and prognostic prediction in various ILDs, including PM/DM-ILD (11, 12). However, the considerable heterogeneity in radiologic features poses significant challenges to standardized interpretation. Recently, the FLAIR clinical prediction model, integrating clinical variables with CT imaging scores, has been developed to improve prognostic accuracy (6). However, its applicability is limited to patients with anti-MDA5-positive DM and has not been validated in anti-MDA5-negative populations.

Therefore, this study aims to develop and validate a prognostic model for acute or subacute progression in patients with PM/DM-ILD by integrating semiquantitative HRCT scoring with key clinical and serologic risk factors using multivariate Cox regression and internal validation.

## 2 Materials and method

### 2.1 Study population

This study retrospectively enrolled consecutive patients with idiopathic PM/DM who underwent HRCT at the Affiliated Hospital of North Sichuan Medical College from January 2020 to December 2024. The Institutional Review Board of our hospital approved the study protocol (Approval No. 2025ER289-1). Written informed consent was waived, considering the retrospective study design. All HRCT examinations and laboratory tests were performed for routine clinical purposes.

**Figure 1:**
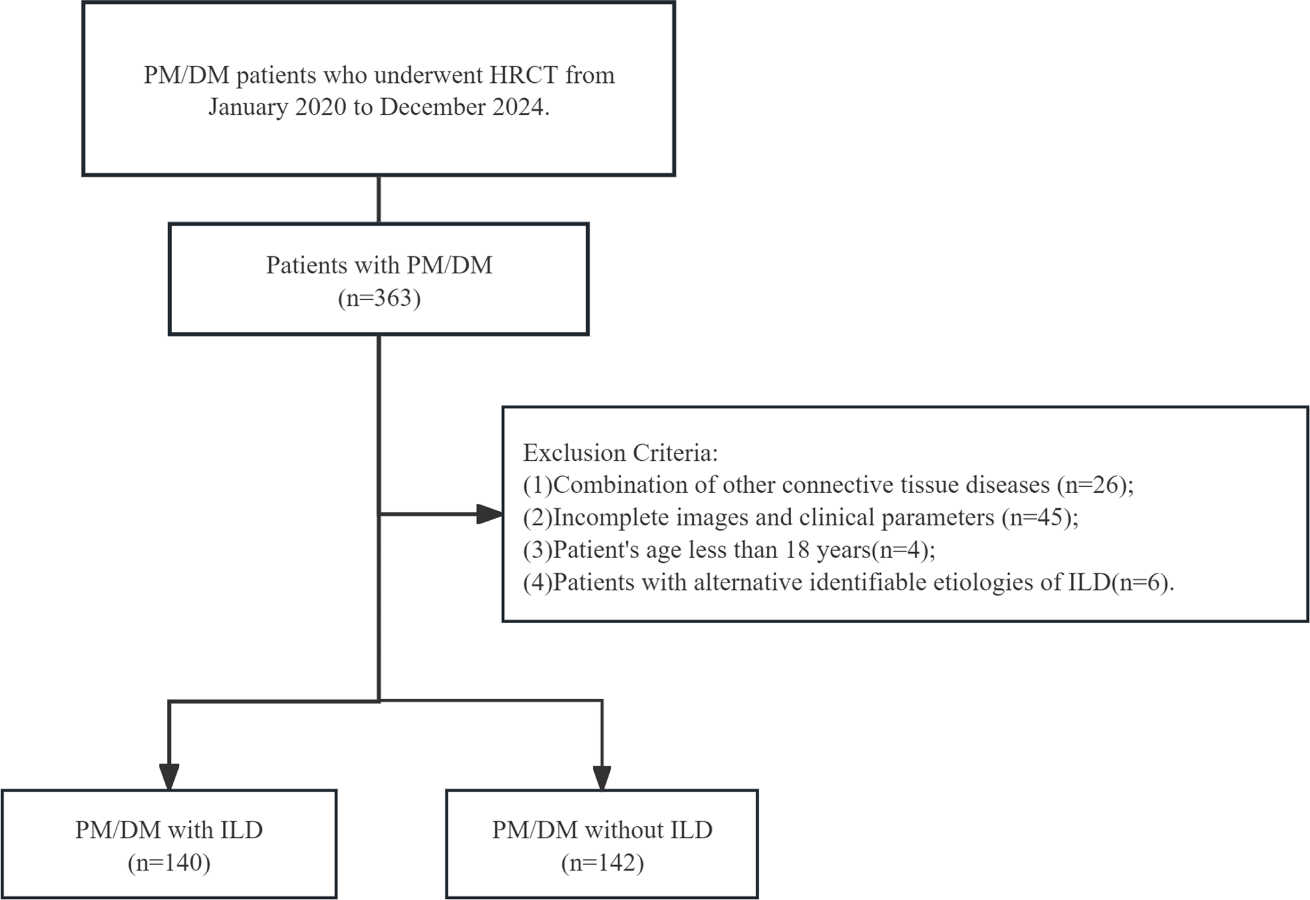
Flowchart illustrating the inclusion and exclusion criteria for the study population.

During the study period, diagnoses in the electronic medical records were coded as polymyositis/dermatomyositis (PM/DM) in routine clinical practice. For this study, PM/DM case classification was standardized using the 2017 European League Against Rheumatism/American College of Rheumatology classification criteria (13). PM/DM-ILD diagnosis and radiologic classification followed the 2013 American Thoracic Society/European Respiratory Society (ATS/ERS) guidelines (14). Inclusion criteria were: (1) age ≥ 18 years; (2) classification within the PM/DM spectrum as defined above, and availability of complete baseline HRCT scans covering the entire lung field from the apices to the diaphragm. Exclusion criteria were: (1) inadequate HRCT image quality or incomplete baseline clinical data; (2) coexistence of other connective tissue diseases; and (3) alternative identifiable etiologies of interstitial lung disease, such as infection, drug-induced pneumonitis, or occupational/environmental exposure–related ILDs (e.g., tuberculosis, bacterial or fungal pneumonia, pneumoconiosis, or radiation-induced lung injury). Cases of suspected infection or drug-induced pneumonitis were excluded based on clinical history, microbiological tests, and radiologic–clinical correlation. Specifically, patients with recent respiratory infection, positive sputum or bronchoalveolar lavage cultures, or a temporal association between HRCT changes and new medication exposure were excluded after multidisciplinary discussion (involving radiologists, pulmonologists, and rheumatologists). A detailed flowchart of the patient selection process and study design is presented in **Figure 1**.

### 2.2 Clinical data collection

At baseline (initial diagnosis and first HRCT at our hospital), comprehensive clinical data were collected, including age, sex, smoking status, alcohol use, hypertension, diabetes mellitus, disease duration and fever. Laboratory evaluations included lactate dehydrogenase (LDH), creatine kinase (CK), CRP, erythrocyte sedimentation rate (ESR), and neutrophil-to-lymphocyte ratio (NLR) measurements. Autoantibody profiles were assessed, comprising anti-MDA5, anti-Ro52, and anti–aminoacyl-tRNA synthetase (anti-ARS) antibodies. Anti-ARS status was categorized as ARS-negative (all tested negative for anti-ARS antibodies), Jo-1 positive, and non–Jo-1 anti-ARS positive (any of PL-7/PL-12/EJ/OJ/KS positive, if tested). Myositis-specific autoantibody testing was conducted at an external reference laboratory (High-Throughput Medical Technology Co., Ltd.). The baseline treatment status at the initial HRCT was extracted from medical records, which encompassed exposure to glucocorticoids, conventional synthetic disease-modifying antirheumatic drugs, Janus kinase inhibitors, alkylating agents, calcineurin inhibitors, biological agents, and antifibrotic agents.

### 2.3 HRCT scans

SOMATOM Force (Siemens Healthineers, Germany), LightSpeed VCT (GE Healthcare, China), or iCT128 (Philips Healthcare, Netherlands) was used for all HRCT examinations. Scans were obtained in the supine position at end-inspiration, covering the lung apices to the bilateral adrenal glands. Scanning parameters included a tube voltage of 120 kV and a tube current of 100 mA. Images were acquired with both 5-mm slice thickness and interslice gap and reconstructed using a thin-slice algorithm to a matrix size of 512 × 512 pixels. All HRCT images were exported in Digital Imaging and Communications in Medicine format from the Picture Archiving and Communication System for further analysis. Raw scan data were transferred to a dedicated postprocessing workstation for thin-slice reconstruction (slice thickness: 1 mm; reconstruction interval: 1 mm).

### 2.4 HRCT data analysis

All HRCT images were exported to a dedicated workstation (UAI Discover - Pneumonia, version 20241030sp1, United Imaging Intelligence, China) for postprocessing analysis, systematically recording relevant radiologic features and semiquantitative scores.

**Figure 2:**
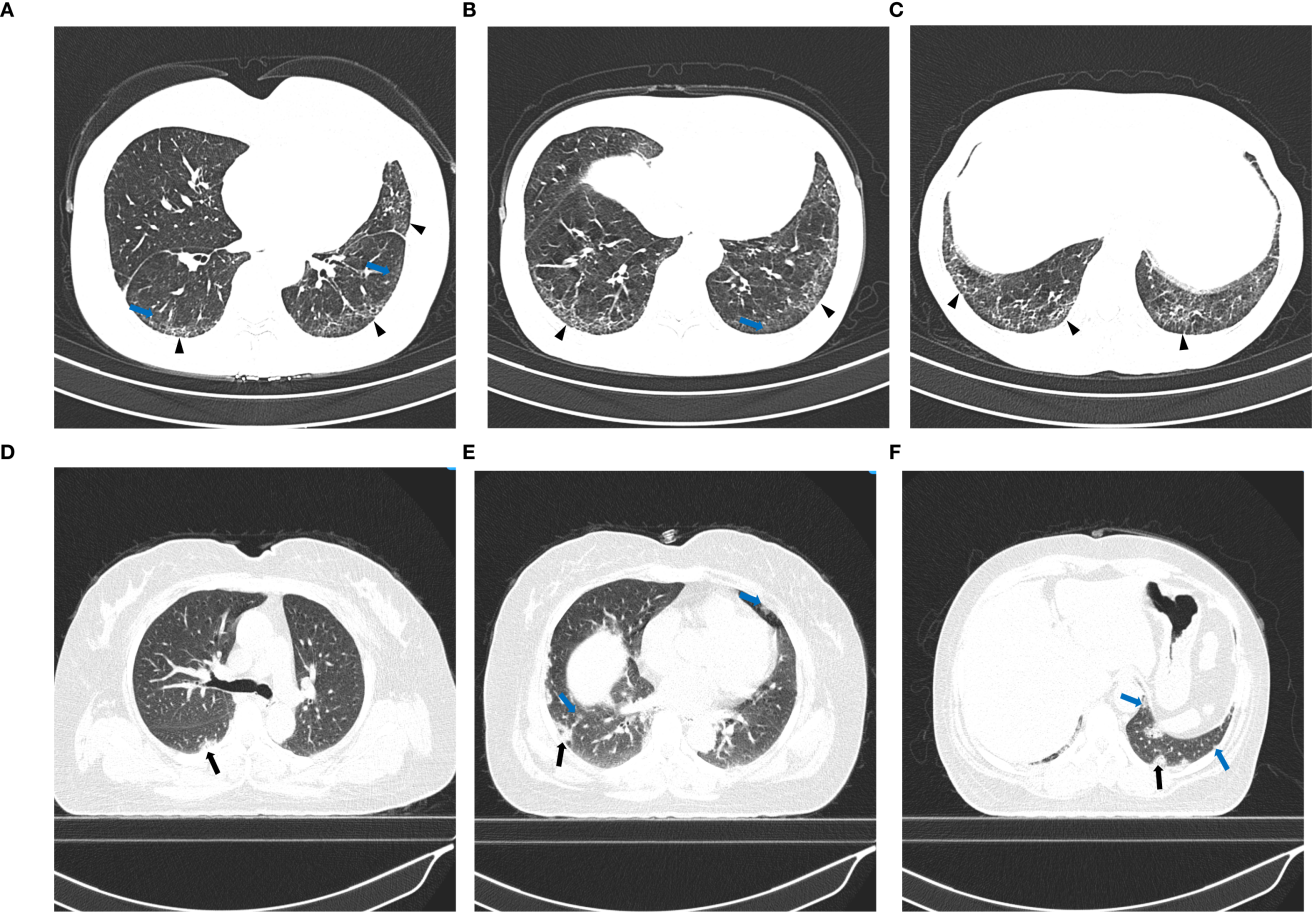
Representative HRCT findings in two patients with PM/DM-ILD. **(A–C)** A 58-year-old man showing an NSIP pattern with ground-glass opacities involving five lung segments and reticular opacities involving seven segments, with a total HRCT score of 9. Blue arrows indicate ground-glass opacities, and black short arrows indicate reticulation. **(D–F)** A 49-year-old woman showing an OP pattern with ground-glass opacities involving five lung segments and consolidation involving five segments, with a total HRCT score of 7. Black arrows indicate consolidation.

The extent of lung involvement was visually scored using the semiquantitative HRCT scoring system described above, and ILD patterns were classified according to the ATS/ERS diagnostic criteria. HRCT scores were identified using Camiciottoli et al.’s semiquantitative visual scoring system (15), assessing pulmonary abnormalities across (1) lesion type: ground-glass opacities (GGO) (1 point), consolidation (2 points), irregular interlobular septal thickening (3 points), reticulation (4 points), and honeycomb cysts (5 points); and (2) lesion extent: 1–3 affected pulmonary segments (1 point), 4–9 segments (2 points), and > 9 segments (3 points). A composite HRCT score of 0–30 was calculated by summing the scores from both domains, with 0 points indicating no detectable abnormalities in any of the five lesion categories. **Table 1** summarizes the details of the HRCT semi-quantitative scoring criteria. **Figure 2** illustrates representative examples of the HRCT semiquantitative scoring in two patients. Two thoracic radiologists (with 3 and 14 years of experience in diagnostic radiology, respectively), blinded to all clinical and laboratory information, independently evaluated all HRCT scans. To evaluate the reproducibility of HRCT scoring, both inter- and intra-observer reliability were assessed. For inter-observer reliability, the intra-class correlation coefficient (ICC) was calculated between the two radiologists’ HRCT scores using a two-way random-effects model with absolute agreement. To assess intra-observer reproducibility, one radiologist re-evaluated HRCT scans from a randomly selected subset of 50 patients after an interval of three months, blinded to the initial results. ICC values greater than 0.80 were considered to indicate excellent agreement. In cases of initial scoring discrepancies between the two radiologists, final scores were determined through consensus review.

**Table 1:** Semi-quantitative HRCT scoring criteria.

Score	Types of pathological manifestations	Distribution of pulmonary lesions.
1	Ground-glass opacification	1–3 pulmonary segments
2	Consolidation	4–9 pulmonary segments
3	Irregular interlobular septal thickening	more than 9 pulmonary segments
4	Reticulation	
5	Honeycomb cysts	

### 2.5 Follow-up

Follow-up data were systematically collected from hospital records and outpatient clinical visits, with a median follow-up duration of 24.71 weeks (IQR: 7.71–25.71 weeks). Follow-up duration was expressed in weeks for improved temporal resolution in survival analysis. Frequency and intensity monitoring were tailored to each patient’s clinical condition. All patients underwent at least two HRCT scans and corresponding clinical assessments. Observation interval was the time from the initial HRCT acquisition to the occurrence of RP-ILD, subacute progressive ILD, or the study’s end date-whichever came first.

RP-ILD and subacute progressive ILD were defined following established diagnostic criteria and identified based on the onset of one or more of the following conditions within 1 or 3 months, respectively, as the onset of respiratory symptoms: (1) hospitalization or oxygen therapy initiation due to acute and progressive respiratory deterioration; (2) HRCT revealing a ≥ 20% increase in the extent of interstitial abnormalities; (3) arterial blood gas analysis revealing respiratory failure or partial pressure of oxygen reduction of > 10 mmHg (14).

### 2.6 Statistical analysis

SPSS version 27.0 (IBM Corp., Armonk, NY, USA) and R statistical software version 4.2.2 (R Foundation for Statistical Computing, Vienna, Austria) were used for statistical analyses. Continuous variables were assessed for normality using the Shapiro–Wilk test and visual inspection of histograms and Q–Q plots. Continuous variables were expressed as mean ± standard deviation or median with interquartile range (IQR) for normally and nonnormally distributed data, respectively. Categorical variables were summarized as counts and percentages. For between-group comparisons, an independent Student’s t-test (or one-way analysis of variance, as appropriate) and the Mann–Whitney U test were used to analyze normally and nonnormally distributed variables, respectively. The chi-square (χ²) test or Fisher’s exact test was used for categorical variable assessment, as appropriate. To explore whether predictors differed between acute and subacute phases, time-window sensitivity analyses were conducted (acute, ≤ 30 days with censoring at day 30; subacute, 31–90 days using a 30-day landmark approach among patient event-free at day 30). Univariate and multivariate logistic regression analyses were conducted to identify risk factors associated with PM/DM-ILD occurrence. The variance inflation factor (VIF) was used for multicollinearity assessment among covariates. Kaplan–Meier survival analysis with log-rank testing was conducted to compare cumulative incidence rates. Univariate Cox proportional hazards regression was employed to explore associations between clinical/imaging factors and ILD progression. To reduce the risk of overfitting associated with automated stepwise procedures, variables for the multivariable Cox model were selected using penalized Cox regression (LASSO) with cross-validation. The predictors selected by LASSO were then refitted in an unpenalized multivariable Cox proportional hazards model to report hazard ratios (HRs), 95% confidence intervals (CIs), and P values. Given that the HRCT-derived risk group was established based on the anti-MDA5 status and the total semiquantitative HRCT score (including component subscores), only the risk group was entered as the imaging predictor in the multivariable model, with its component variables excluded simultaneously to prevent redundancy. The concordance index (C-index), with higher values indicating better predictive accuracy, was used to evaluate the discriminative performance of the algorithm. To estimate optimism and derive an optimism-corrected C-index, internal validation was performed by bootstrap resampling (1,000 iterations). Further, the integrated discrimination improvement (IDI) and the net reclassification improvement (NRI) indices were used to assess the reclassification performance of the algorithm. Algorithm performance was assessed using calibration plots to evaluate agreement between predicted and observed risks, and decision curve analysis (DCA) to evaluate clinical net benefit. A two-sided P-value of < 0.05 indicated statistical significance.

## 3 Results

### 3.1 Baseline characteristics

Of the 282 patients with PM/DM (mean age: 53.4 ± 13.2 years), 191 (67.9%) were female. Of them, 140 (49.65%) patients were identified with ILD (mean age: 55.6 ± 12.4 years), 71.0% of whom were female. **Supplementary Tables S1**, **S2** summarizes baseline characteristics. Multivariate logistic regression analysis identified older age, smoking history, anti-MDA5 antibody positivity, and anti-Ro-52 antibody positivity as independent risk factors for ILD in patients with PM/DM.

### 3.2 Association of clinical indicators and HRCT imaging with RP/subacute progression.

The predominant HRCT imaging patterns in the 140 patients with PM/DM-ILD included NSIP in 78 (55.7%), followed by a mixed NSIP–OP pattern in 33 (23.6%), OP in 19 (13.6%), UIP in 7 (5.0%), and DAD in 3 (2.1%) patients. Inter-observer reliability of the HRCT scoring system was high, with an ICC of 0.893 between the two radiologists. Intra-observer reproducibility, assessed by repeated evaluation of 50 randomly selected patients after a 3-month interval, also showed strong agreement (ICC = 0.901). During a median follow-up period of 24.71 weeks (IQR: 7.71–25.71 weeks), 56 (40.0%) patients experienced progressive ILD. Among those with disease progression, 28 (50.0%) and 28 (50.0%) patients demonstrated acute and subacute progressions, respectively. **Table 2** summarizes the baseline characteristics of the study population. Compared with the stable group, patients in the PM/DM-ILD progression group were significantly older (P < 0.001) and more likely to present with fever at initial diagnosis (P = 0.002), and had a shorter disease duration (P = 0.038). The progression group also had markedly higher positivity rates of anti-MDA5 antibodies (53.6% vs. 20.2%, P < 0.001) and anti-Ro52 antibodies (56.0% vs. 39.3%, P < 0.001) than the non-ILD group. In addition, HRCT features, including scores for ground-glass opacities, consolidation, and interlobular septal thickening, were significantly higher in the progression group compared with the stable group (P < 0.05). The baseline treatment exposure at the initial HRCT was broadly comparable between groups, and no significant differences were found across treatment categories (all P > 0.05). Notably, Janus kinase inhibitors were only used in the progression group (0.0% vs. 5.4%), showing a borderline difference (P = 0.062). No significant differences were observed in the remaining variables between the two groups (P > 0.05).

**Table 2:** Baseline of clinical characteristics between the PM/DM-ILD progressive group and PM/DM-ILD stable group.

Variable	Total (n=140)	Progressive group (n=56)	Stable group (n=84)	P value
Clinical characteristics				
Age(y)	56.1 ± 11.8	59.4 ± 8.7	54.4 ± 13.0	<0.001
Gender (female), n (%)	101 (72.1)	42 (75.0)	59 (70.2)	0.481
Hypertension, n (%)	22 (15.7)	10 (17.9)	12 (14.3)	0.680
Diabetes mellitus, n (%)	11 (7.9)	6 (10.7)	5 (6.0)	0.386
Smoking, n (%)	41 (29.3)	18(32.1)	23 (27.4)	0.784
Alcohol, n (%)	26 (18.6)	12 (21.4)	14 (16.7)	0.840
Fever, n (%)	40 (28.6)	24 (42.9)	16 (19.0)	0.002
Disease duration, days	56.50 (8.00–246.00)	69.50 (8.00–384.50)	41.50 (11.00–83.00)	0.038
Laboratory indicators				
LDH, (U/L)	308.0 (224.0-465.0)	378.5 (240.0-536.8)	297.0 (215.5-417.5)	0.135
CK, (U/L)	159.0 (45.0-1511.0)	295.0 (43.8-2361.3)	155.0 (45.2-1279.0)	0.283
CRP, (mg/L)	5.8 (1.5-18.4)	5.1 (1.2-17.5)	5.8 (1.4-20.3)	0.979
ESR, (mm/h)	31.0 (16.0-50.0)	31.5 (19.0-49.3)	33.0 (17.5-53.0)	0.764
NLR	4.2 (2.6-6.2)	4.5 (2.9-6.0)	3.7 (2.5-6.5)	0.528
Autoantibody information				
anti-MDA5 antibody	47 (33.6)	30 (53.6)	17 (20.2)	<0.001
anti-ARS antibody	46 (32.9)	17 (30.4)	29 (34.5)	0.607
anti-Ro52 antibody	69 (49.3)	47(56.0)	22 (39.3)	<0.001
Baseline treatment				
GCs	129 (92.1)	78 (92.9)	51 (91.1)	0.700
csDMARDs	80 (57.1)	50 (59.5)	30 (53.6)	0.486
JAKi	3 (2.1)	0 (0.0)	3 (5.4)	0.062
Alkylating agents	54 (38.6)	30 (35.7)	24 (42.9)	0.395
CNIs	9 (6.4)	5 (6.0)	4 (7.1)	1.000
bDMARDs	4 (2.9)	1 (1.2)	3 (5.4)	0.302
Antifibrotic agents	28 (20.0)	18 (21.4)	10 (17.9)	0.605
HRCT imaging pattern				
OP pattern, n (%)	19(13.6)	9(16.1)	10(11.9)	0.481
HRCT scores				
Ground-glass opacification	3.0(2.0-3.0)	3.0(2.0-4.0)	3.0(0.0-3.0)	<0.001
Consolidation	0.0(0.0-4.0)	1.5(0.0-4.0)	0.0(0.0-3.0)	0.019
Irregular interlobular	0.0(0.0-3.0)	0.0(0.0-4.0)	0.0(0.0-0.0)	0.032
septal thickening	5.0(5.0-6.0)	6.0(5.0-7.0)	6.0(5.5-6.0)	0.690
Reticulation	0.0(0.0-0.0)	0.0(0.0-0.0)	0.0(0.0-0.0)	0.702
Total scores	10.0(8.0-12.0)	14.3(11.0-16.0)	9.0(7.5-14.0)	<0.001

PM/DM-ILD, Polymyositis and dermatomyositis associated with interstitial lung disease; LDH, lactate dehydrogenase; CK, creatine kinase; CRP, C-reactive protein; ESR, erythrocyte sedimentation rate; NLR, neutrophil-to-lymphocyte ratio; anti-MDA5 antibody, anti-melanoma differentiation-associated gene 5 antibodies; anti-Jo1 antibody, anti-histidyl-tRNA synthetase autoantibody; GCs, glucocorticoids; csDMARDs, conventional synthetic disease-modifying antirheumatic drugs; JAKi, Janus kinase inhibitors; CNIs, calcineurin inhibitors; bDMARDs, biologic disease-modifying antirheumatic drugs.

**Table 3** shows the univariate and multivariate Cox proportional hazards analyses. Univariate Cox regression revealed that older age, higher CK levels, fever at initial presentation, shorter disease duration, OP pattern, and anti-MDA5 antibody positivity were significantly associated with ILD progression (all P < 0.05). Both a higher total semiquantitative HRCT score (*P* = 0.007) and an increased GGO score (*P* = 0.025) were significantly associated with the risk of acute/subacute ILD progression. In the time-to-event analyses of baseline treatment categories, no medication class showed a significant association with progression; however, the baseline use of Janus kinase inhibitors showed a non-significant trend toward a higher risk (HR 2.95, 95% CI 0.92–9.47; P = 0.069). All VIF values were < 5, indicating no significant multicollinearity among the included variables. Using penalized Cox regression (LASSO) with cross-validation for variable selection, the retained predictors were refitted in an unpenalized multivariable Cox proportional hazards model to obtain HR, 95% CIs, and P values. In the refitted model, older age (HR = 1.03, 95% CI 1.00–1.05, P = 0.036), fever at initial presentation (HR = 1.96, 95% CI 1.14–3.37, P = 0.015), CK (HR = 1.00, 95% CI 1.00–1.00, P = 0.024), anti-MDA5 antibody (HR = 4.82, 95% CI 2.65–8.77, P < 0.001), and HRCT total scores (HR = 1.20, 95% CI 1.11–1.30, P < 0.001) were independently associated with acute or subacute PM/DM-ILD progression.

**Table 3:** Univariate and multivariate cox regression analysis of risk factors for disease progression in patients with PM/DM-ILD.

Variable	Univariate cox analysis		Multivariable cox analysis	
	HR (95%CI)	P value	HR (95%CI)	P value
Clinical characteristics				
Age(y)	1.03 (1.01-1.05)	0.018	1.03 (1.00–1.05)	0.036
Gender (female), n(%)	0.82 (0.44-1.52)	0.519		
Hypertension, n (%)	1.05 (0.50-2.22)	0.895		
Diabetes mellitus, n (%)	1.43 (0.68-3.02)	0.350		
Smoking, n (%)	0.89 (0.44-1.81)	0.744		
Alcohol, n (%)	1.05 (0.50-2.22)	0.898		
Fever, n (%)	2.30 (1.35-3.91)	0.002	1.96 (1.14–3.37)	0.015
Disease duration	1.00 (1.00-1.00)	0.047	–	–
Laboratory indicators				
LDH, (U/L)	1.00 (1.00-1.00)	0.049	–	–
CK, (U/L)	1.00 (1.00-1.00)	0.030	1.00 (1.00-1.00)	0.030
CRP, (mg/L)	1.00 (0.99-1.01)	0.631		
ESR, (mm/h)	1.00 (1.00-1.01)	0.526		
NLR	0.98 (0.94-1.04)	0.556		
Autoantibody information				
anti-MDA5 antibody	3.00 (1.77-5.18)	<0.001	4.82 (2.65–8.77)	<0.001
anti-ARS antibody	0.86 (0.49–1.52)	0.602		
anti-Ro52 antibody	0.63 (0.39-1.06)	0.082		
Baseline treatment				
GCs	0.96 (0.38-2.42)	0.939		
csDMARDs	0.86 (0.51-1.46)	0.583		
JAKi	2.95 (0.92-9.47)	0.069		
Alkylating agents	1.24 (0.73-2.10)	0.433		
CNIs	1.09 (0.39-3.01)	0.870		
bDMARDs	2.42 (0.75-7.78)	0.138		
Antifibrotic agents	0.84 (0.42-1.66)	0.612		
HRCT imaging pattern				
OP pattern	1.32 (1.05-1.72)	0.042		
HRCT scores				
Ground-glass opacification	1.48 (1.16-1.87)	0.001	1.33 (1.05–1.67)	0.018
Consolidation	1.21 (1.06-1.39)	0.005	1.23 (1.07–1.42)	0.005
Irregular interlobular	1.16 (1.03-1.30)	0.014	1.13 (1.00–1.28)	0.046
septal thickening	0.96 (0.86-1.07)	0.960		
Reticulation	1.02 (0.5 -1.24)	0.810		
Total scores	1.17 (1.09-1.26)	<0.001	1.20 (1.11–1.30)	<0.001
Groups				
Low-risk group		<0.001		<0.001
Mediate-risk group	4.53 (1.99-10.29)	<0.001	4.45 (1.95-10.17)	<0.001
High-risk group	9.93 (4.12-23.90)	<0.001	11.81 (4.83-28.85)	<0.001

HR, Hazard Ratio; 95% CI, 95% Confidence Interval; PM/DM-ILD, Polymyositis and dermatomyositis associated with interstitial lung disease; LDH, lactate dehydrogenase; CK, creatine kinase; CRP, C-reactive protein; ESR, erythrocyte sedimentation rate; NLR, neutrophil-to-lymphocyte ratio; anti-MDA5 antibody, anti-melanoma differentiation-associated gene 5 antibodies; anti-Jo1 antibody, anti-histidyl-tRNA synthetase autoantibody; OP, organizing pneumonia; GCs, glucocorticoids; csDMARDs, conventional synthetic disease-modifying antirheumatic drugs; JAKi, Janus kinase inhibitors; CNIs, calcineurin inhibitors; bDMARDs, biologic disease-modifying antirheumatic drugs.

**Figure 3:**
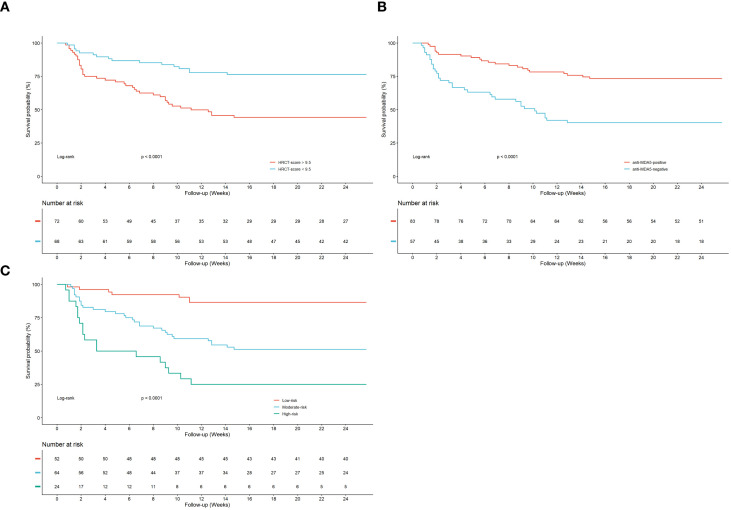
**(A)** Progression-free survival rate and Kaplan–Meier survival curves based on HRCT total score thresholds, categorized as ≤9.0 or >9.0 HU. **(B)** Progression-free survival rate and Kaplan–Meier survival curves based on anti-MDA5 antibody status, categorized as anti-MDA5 antibody-positive or anti-MDA5 antibody-negative. **(C)** Stratified cumulative survival curves based on HRCT total score thresholds and anti-MDA5 antibody status, categorized into low-, medium-, and high-risk groups.

Receiver operating characteristic (ROC) curve analysis was conducted to assess the predictive value of the total HRCT score for identifying acute or subacute progression in patients with PM/DM-ILD. The optimal cutoff value was 9.0, generating an area under the curve (AUC) of 0.702 (95% CI, 0.617–0.786), with sensitivity and specificity of 74.2% and 60.6%, respectively, based on the maximum Youden index. This cutoff value of 9.0 was further internally validated using 1,000 bootstrap resamples, confirming its robustness and stability as the optimal threshold. Kaplan–Meier survival analysis revealed that anti-MDA5 antibody positivity (log-rank *P* < 0.001) and increased HRCT scores (≤9.0; log-rank *P* < 0.001) were significantly associated with a higher risk of ILD progression. The corresponding survival curves indicated clear separation between high-risk and low-risk subgroups (**Figures 3A, B**).

**Figure 4:**
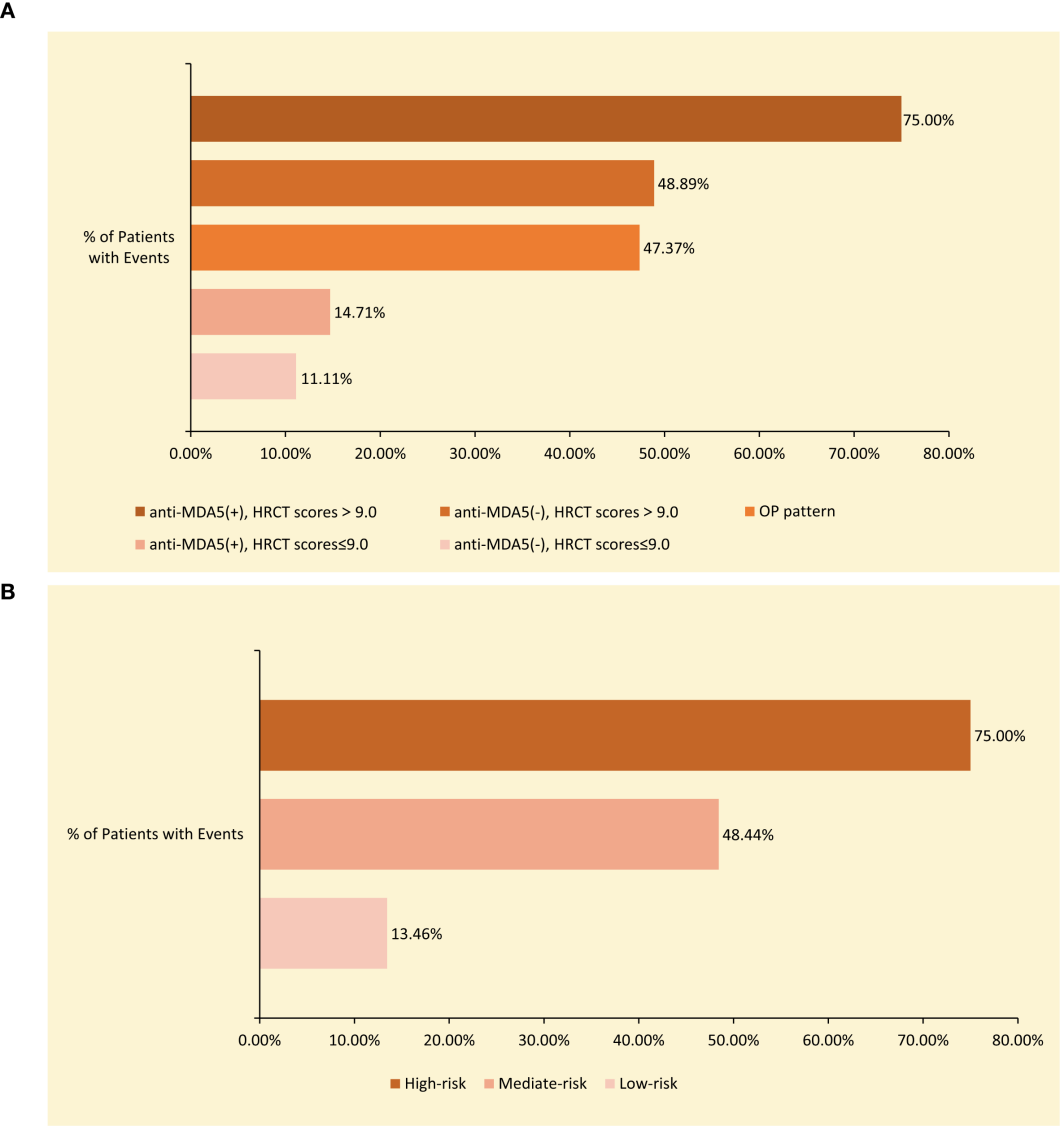
**(A)** Event rates for each of the five subgroups: anti-MDA5(+), HRCT >9.0, anti-MDA5(-), HRCT >9.0, OP pattern, anti-MDA5(-), HRCT <9.0, and anti-MDA5(+), HRCT ≤9.0. **(B)** Event rates stratified by composite risk groups (high-risk, intermediate-risk, low-risk) based on the integrated prognostic algorithm.

According to the Kaplan–Meier survival analysis stratified by the HRCT score threshold of 9.0 and anti-MDA5 antibody status, patients with PM/DM-ILD were initially categorized into (1) anti-MDA5-negative with HRCT score of ≤9.0, (2) anti-MDA5-positive with HRCT score of ≤9.0, (3) anti-MDA5-negative with HRCT score of > 9.0, and (4) anti-MDA5-positive with HRCT score of > 9.0. Notably, despite relatively low baseline HRCT scores, patients with an OP pattern demonstrated a higher incidence of acute or subacute ILD progression (9 of 19 patients), prompting the inclusion of the OP pattern as an additional stratification factor, thereby generating five subgroups. Thereafter, a novel risk stratification algorithm was developed according to three key variables: HRCT score (cutoff, 9.0), presence of OP pattern, and anti-MDA5 antibody status. This algorithm effectively categorized patients into three distinct prognostic risk groups. **Figure 4** illustrates event rates across the five subgroups and three risk groups.

- Low-risk: anti-MDA5-negative with HRCT scores of ≤9.0 (event rate: 11.11%) and anti-MDA5-positive with HRCT scores of ≤9.0 (event rate: 14.71%);
- Mediate-risk: anti-MDA5-negative with HRCT scores of > 9.0 (event rate: 48.89%) and OP pattern (event rate: 47.37%);
- High-risk: anti-MDA5-positive with HRCT scores of > 9.0 (event rate: 75.00%).

Kaplan–Meier analysis with log-rank testing revealed a significantly higher risk of disease progression in the mediate- and high-risk groups compared with the low-risk group (log-rank *P* < 0.001; **Figure 3C**). The HRCT-derived risk group was further assessed to examine whether progression risk increased across clinically defined strata. Specifically, a stepwise escalation was noted in hazard across prognostic strata: compared with the low-risk group, the intermediate-risk group exhibited a significantly increased risk of progression (HR = 4.45, 95% CI 1.95–10.17, P < 0.001), and the high-risk group showed the strongest association (HR = 11.81, 95% CI 4.83–28.85, P < 0.001). Time-window sensitivity analyses were also performed, which separated acute (≤30 days) and subacute (31–90 days) progression using a 30-day landmark approach. The HRCT-based risk group demonstrated consistent association with progression in both windows, with age and fever showing relatively stronger associations in the subacute phase (**Supplementary Table S4**).

**Figure 5:**
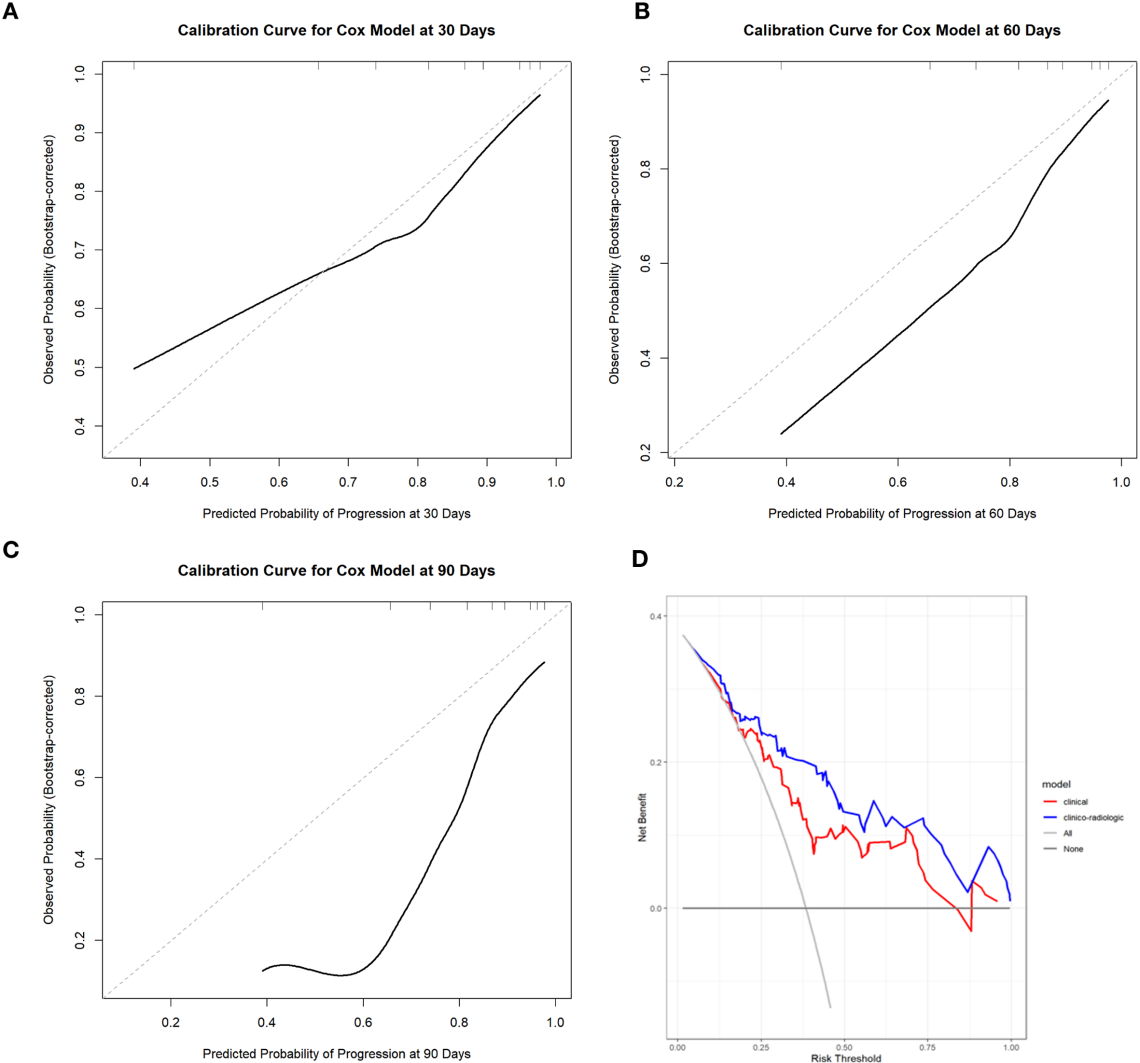
Calibration and decision curve analyses of the clinico-radiologic algorithm. **(A–C)** Time-dependent calibration curves at 30 days **(A)**, 60 days **(B)**, and 90 days **(C)** showing agreement between predicted and observed risks (dashed line indicates ideal calibration). **(D)** Decision curve analysis demonstrating that the clinico-radiologic algorithm yields a higher net benefit than the clinical algorithm across most threshold probabilities.

Following these findings, we developed a prognostic algorithm integrating clinical risk factors, semiquantitative HRCT scores, and the presence of an OP pattern to predict acute or subacute progression in patients with PM/DM-ILD. The incorporation of the three-tiered risk stratification (high, intermediate, and low) improved discrimination, which increased the apparent C-index from 0.642 to 0.764, and bootstrap validation (1,000 resamples) yielded an optimism-corrected C-index of 0.751. The results revealed that compared to the algorithm including only clinical factors, the integration of the composite algorithm for risk stratification significantly improved risk reclassification, with an IDI 0.218 (95% CI: 0.093-0.347) and an NRI 0.470 (95% CI: 0.257-0.621). Calibration plots showed good agreement between predicted and observed risks. Calibration plots at 30 and 60 days demonstrated generally good agreement between predicted and observed risks, whereas calibration at 90 days deviated more noticeably from the ideal line, indicating reduced calibration stability at the longer horizon. Results of the decision curve analysis indicated that the integrated clinicoradiological algorithm provided consistently greater net benefit across a wide range of threshold probabilities compared with clinical factors alone, supporting its potential clinical utility (**Figure 5**). The comparison of the C-index, Chi-square statistic, relative IDI, and category-free NRI among different models is summarized in **Supplementary Table S5**.

## 4 Discussion

In this study, multivariate logistic regression analysis identified smoking history, anti-MDA5 antibody positivity, and Ro-52 antibody positivity as independent risk factors for ILD, a complication in patients with PM/DM. Further, older age, fever at initial presentation, anti-MDA5 antibody positivity, higher GGO scores, and increased total HRCT scores were independently associated with ILD progression. To assess the risk of rapid or subacute ILD progression in patients with PM/DM, this study developed a predictive algorithm based on semiquantitative HRCT scoring, clinical characteristics, and serological markers. The algorithm showed promising discriminatory ability in our cohort, indicating its use in early risk stratification and clinical management.

Acute or subacute ILD progression in patients with PM/DM-ILD represents a serious and potentially life-threatening clinical entity (16). Among these, RP-ILD is a particularly aggressive phenotype, characterized by abrupt clinical deterioration, including worsening hypoxemia, rapidly progressive radiological abnormalities (e.g., extensive GGO or consolidation), and the development of respiratory failure within a few weeks of symptom onset (13, 17). This condition has a high short-term mortality rate, particularly in patients positive for anti-MDA5 antibodies (18). Subacute ILD progression is less fulminant than RP-ILD; however, it is increasingly considered a prodromal or subclinical form of the rapidly progressive variant. Early manifestations are frequently nonspecific (e.g., mimicking infection or drug-related pneumonitis), thereby delaying diagnosis and appropriate management (19, 20). Without timely recognition and intervention, subacute progression may advance to classical RP-ILD (21). Moreover, previous studies have revealed that severe pulmonary complications persist in patients with subacute progression who are anti-MDA5-negative but demonstrate abnormal imaging features and increased inflammatory markers (22). Given these clinical considerations, all patients who showed ILD progression within 0–3 months of initial assessment were categorized into the progression group, regardless of course classification, either acute or subacute.

Current evidence indicates multiple clinical parameters as predictors for ILD development in patients with PM/DM. For instance, increased ESR and CK levels and advanced age have been associated with a higher risk of ILD onset (8). In our study, advanced age, smoking history, and anti-MDA5 were identified as independent predictors of ILD development in patients with PM/DM, consistent with findings from previous studies (23). Notably, the anti-MDA5 antibody (a myositis-specific autoantibody) is not only a significant risk factor for ILD onset in PM/DM but also a crucial disease progression and mortality predictor among patients with PM/DM-ILD (24), anti-MDA5 positivity is characterized by an exaggerated type I interferon signature, and RNA-containing immune complexes formed by MDA5 and its autoantibodies can trigger IFN-α production via endosomal TLR pathways; together with monocyte–macrophage activation in the lung, this biology provides a plausible link to the acute/subacute progression phenotype (25). Baseline treatment exposure at initial HRCT was broadly comparable between the stable and progression groups, and no medication class showed a significant association with progression in the time-to-event analyses. Notably, Janus kinase inhibitors were used only in the progression group and appeared numerically higher in relation to progression risk; however, very few patients were exposed, resulting in wide CIs. Given the retrospective design and nonstandardized treatment timing and dosing, residual treatment-related confounding cannot be fully excluded; thus, larger prospective studies with standardized regimens are warranted.

In recent years, HRCT has become a crucial tool for accurately assessing both the pattern and extent of ILD, providing valuable insights into its pathological characteristics (26). Thus, quantitative and semiquantitative HRCT scoring systems have been important prognostic tools for evaluating disease severity and predicting clinical outcomes in patients with ILD. In our study, a higher baseline total HRCT score and increased GGO score were significantly associated with disease progression. A higher HRCT score reflects extensive inflammatory infiltration, alveolar exudation, and early fibroproliferative activity, processes that are pathophysiologically linked to rapid parenchymal damage and impaired gas exchange (27). The pathophysiological significance of GGO, which are known to reflect alveolar septal infiltration by inflammatory cells, alveolar wall edema, or intra-alveolar exudation, may explain this association. These findings indicate an active inflammatory phase of ILD, which, if not adequately controlled, may rapidly advance into irreversible pulmonary fibrosis (28). Furthermore, GGO is often accompanied by traction bronchiectasis, indicating concomitant architectural distortion and early fibrotic remodeling. These lesions are frequently less responsive to immunosuppressive therapy and are commonly associated with impaired pulmonary diffusing capacity, persistent hypoxemia, and increased risk of respiratory failure (29). These observations are consistent with previous reports. For instance, Xu et al. (30) employed the HRCT scoring method, originally proposed by Kazerooni et al. (31), to assess GGO and consolidations, and revealed that increased GGO and consolidation scores were independent predictors of mortality in patients with anti-MDA5 antibody-positive DM. Similarly, Liu et al. (17) reported that in a 6-month follow-up cohort of 214 patients with anti-MDA5-positive DM, higher GGO scores were independently associated with early mortality.

Our analysis further incorporated a composite HRCT scoring system to simultaneously assess the extent and severity of GGO, pulmonary consolidation, interlobular septal thickening, reticular changes, and their distribution across the lung lobes. This semiquantitative scoring approach enables a comprehensive disease burden assessment on imaging. Similar HRCT scoring systems have previously been employed to predict survival outcomes in patients with systemic sclerosis-associated ILD, demonstrating their prognostic value in connective tissue disease-related ILD (15). This approach enabled a more comprehensive quantification of pulmonary abnormalities, potentially improving the predictive accuracy for disease progression in patients with PM/DM-ILD.

Notably, patients with the OP pattern demonstrated relatively low baseline HRCT scores (mean: 8.2). However, a substantial proportion of patients with the OP pattern exhibited acute or subacute ILD progression, approaching one-half of this subgroup (9/19, 47.4%). This observation may appear inconsistent with the commonly accepted notion that OP tends to be more responsive to corticosteroids and is often associated with better outcomes than UIP or fibrotic NSIP. A key consideration is that our endpoint focused on early deterioration within an acute or subacute time window, whereas previous studies supporting a favorable prognosis for OP often assessed longer-term outcomes and were often based on histopathological or multidisciplinary classifications. In PM/DM-ILD setting, an HRCT-defined OP pattern may represent an OP-like, consolidation-predominant inflammatory phenotype that can fluctuate over a short horizon and, in some patients, accompany early clinical deterioration even with limited baseline radiologic extent. We hypothesize that rapid accumulation of inflammatory exudates and fibroblasts within alveolar spaces and terminal bronchioles in OP may impair gas exchange and contribute to short-term worsening (32). In addition, OP identified on transbronchial biopsy in inflammatory myopathies was reported to precede diffuse alveolar damage and poor outcomes in rapidly progressive cases, suggesting that OP-like changes can coexist with, or evolve into, a more aggressive acute lung injury process in this disease context (33). Previous studies have highlighted the prognostic relevance of HRCT patterns in PM/DM-ILD. Enomoto et al. (34) reported that, compared with NSIP, OP was associated with more extensive parenchymal consolidation and a higher frequency of subacute progression. Furthermore, OP features on HRCT, particularly in conjunction with anti-PL-7 antibody positivity, have been linked to an increased risk of developing RP-ILD (35). Given the potential overlap between OP pattern and ASS/anti-ARS positivity, additional anti-ARS-stratified analyses and sensitivity Cox specifications were performed (**Supplementary Table S3**). In these analyses, the association of the OP pattern with progression was attenuated and did not remain significant after accounting for anti-ARS categories. This find highlights that the OP signal may partly reflect underlying serologic or phenotypic heterogeneity and that estimates are sensitive to model specification and sample size. Overall, our multivariable analysis did not support OP patterns as independent predictors in the final model; nevertheless, qualitative pattern recognition remains clinically useful for early risk appraisal, closer short-term monitoring, and timely reassessment of treatment in patients with suspected worsening.

We developed a novel risk stratification algorithm that demonstrated favorable discriminatory performance in predicting disease progression by integrating the OP pattern, total HRCT score, and anti-MDA5 antibody status into a three-tiered composite risk group (high, intermediate, and low). In the refitted Cox model after LASSO selection, the high-risk group demonstrated an 11.81-fold higher risk of disease progression than the low-risk group, whereas the intermediate-risk group exhibited a 4.45-fold higher risk. This stratification approach facilitates early identification of patients at intermediate and high risk, and may support closer monitoring and timely reassessment of immunosuppressive therapy. Moreover, the refined risk algorithm enables further subdivision between intermediate- and high-risk categories, providing a framework for individualized treatment planning. Overall, the integration of clinical, serologic, and imaging variables into a unified risk algorithm yielded good predictive performance for disease progression in PM/DM-ILD, with an apparent Harrell’s C-index of 0.764 and an optimism-corrected C-index of 0.751 after bootstrap internal validation (1,000 resamples). Because the present outcomes encompassed acute and subacute progression within 1–3 months, calibration was evaluated across 30, 60, and 90 days to reflect the clinically relevant time window. Calibration appeared more stable at 30 and 60 days, whereas the 90-day curve demonstrated greater deviation from the ideal line. This pattern may reflect increased censoring and fewer patients remaining under observation at later time points, which can reduce the stability of nonparametric calibration estimates even after bootstrap correction. In addition, treatment regimens and clinical status may change over a 3-month period, and baseline-only prediction model cannot capture such time-varying interventions, potentially contributing to attenuation in calibration at 90 days.

We observed that disease duration tended to be shorter among patients who experienced early progression. This pattern may reflect that some individuals undergo baseline assessment during a more rapidly evolving phase of disease, rather than implying that timing itself drives risk. After multivariable adjustment, the contribution of disease duration was attenuated, supporting the interpretation that its prognostic information is largely shared with baseline clinical severity and radiologic burden, instead of providing strong independent separation between an “early aggressive” and a “late stable” trajectory.

In our stratification framework, anti–MDA5–positive patients with lower baseline HRCT scores appeared to have fewer short-term progression events within the early follow-up window. This observation should be interpreted in terms of baseline radiologic burden and the limited time horizon captured by the model. In MDA5+ DM-ILD, a relatively low HRCT score at presentation does not necessarily indicate biologically quiescent disease, because hyperinflammation and physiologic decline may precede overt radiographic escalation. Accordingly, this subgroup should not be labeled as clinically low risk; instead, it highlights a scenario in which imaging severity may lag behind underlying disease activity. Clinical management should therefore continue to emphasize close monitoring and timely treatment escalation guided by symptoms, oxygenation, and inflammatory biomarkers, even when initial imaging abnormalities appear limited. Therefore, predictions at longer horizons should be interpreted with caution and warrant validation in larger cohorts with standardized follow-up.

Several prognostic models, including FLAIR and STRAD models, were previously proposed to stratify risk among patients with PM/DM who are positive for anti-MDA5 antibodies (6, 36). These models have demonstrated use in identifying high-risk individuals within the anti-MDA5-positive PM/DM-ILD population. Considering these frameworks, we further extended the risk stratification method by incorporating patients with anti-MDA5 antibody negativity and integrating the OP pattern. This expansion enabled a broader applicability of the algorithm across a more heterogeneous PM/DM-ILD population.

In our cohort of 56 patients who experienced disease progression, 22 (39.3%) were negative for anti-MDA5 antibodies. This finding indicates that a substantial proportion of ILD progression occurs in patients without anti-MDA5 antibody positivity, requiring broader risk assessment strategies beyond serological profiling alone. Unlike other studies, patients in our cohort with positive anti-MDA5 antibodies but had low HRCT scores were classified into the low-risk group. This classification is justified because patients with lower HRCT scores generally demonstrate milder radiographic findings, indicating more limited pulmonary involvement. Consequently, according to these HRCT scores, such patients are reasonably categorized into a lower-risk group. Anti-MDA5 antibody positivity is typically associated with severe ILD progression; however, patients with no apparent fibrosis or significant pathological changes on HRCT may be in the early or stable disease phase, where rapid deterioration is unnecessarily imminent. Wang et al (37) classified patients who are anti-MDA5-positive with low HRCT scores were classified as low-risk, as they demonstrated no clear signs of acute or subacute progression and were likely in a relatively stable disease phase.

The developed risk stratification system supports the implementation of personalized management strategies for patients with PM/DM-ILD. Patients with low risk may be spared from excessive immunosuppressive therapy exposure, thereby reducing the likelihood of treatment-related adverse events. In contrast, patients with intermediate and high risks may benefit from early initiation of intensive combination immunosuppressive regimens. Moreover, predictive algorithm that integrate imaging features with clinical variables demonstrated improved discriminatory performance, may assist in identifying patients who could benefit from early intervention.

This study has several limitations. First, this retrospective, single-center, imaging-based cohort study has a relatively limited sample size; inclusion required an evaluable baseline HRCT, and some patients were excluded because of incomplete baseline data. Consequently, selection bias and limited statistical power cannot be fully avoided, and our findings may be most applicable to patients with PM/DM receiving HRCT evaluation in routine practice. Second, although cases within a PM/DM-spectrum framework were re-adjudicated using contemporary criteria and the terminology was revised accordingly, residual misclassification remains possible because reclassification was constrained by the completeness of archived records and available phenotyping at the time of care. In addition, OP cases and antibody-defined subgroups were small, resulting in wide CIs and limiting the ability to detect modest independent effects or interactions. Third, a semiquantitative visual HRCT scoring system originally described in systemic sclerosis–associated ILD was used to standardize extent-based assessment of key abnormalities; although practical for retrospective analyses, this approach has not been specifically validated in PM/DM-ILD and could be further corroborated using PM/DM-tailored scoring frameworks and/or automated quantitative CT methods; additionally, disease duration was retrospectively ascertained and may be imprecise, such that it may not reliably distinguish “early aggressive” from “late stable” presentations, particularly in anti-MDA5–positive disease where radiographic extent may lag behind hyperinflammatory activity. Fourth, external validation was not performed, and correlations with pulmonary function tests (e.g., FVC and DLCO) or longitudinal biomarker changes were not assessed. Therefore, larger multicenter prospective studies with standardized adjudication, comprehensive myositis antibody panels, and independent validation are warranted to refine and confirm the prognostic utility of HRCT-based risk stratification in PM/DM-ILD.

## Data Availability

The raw data supporting the conclusions of this article will be made available by the authors, without undue reservation.
