# Supplementary material for: Prognostic value of HRCT-based risk stratification for acute/subacute progression in polymyositis/dermatomyositis-associated interstitial lung disease
Source: Front Immunol. 2026 Jan 28;17:1748191. doi: 10.3389/fimmu.2026.1748191 (PMC12891071; doi:10.3389/fimmu.2026.1748191)
Supplement: Supplementary file 1 [file SupplementaryFile1.docx]

Supplementary Material

# Supplementary Tables

| **Table S1.** Baseline of clinical characteristics between the PM/DM-ILD group and PM/DM-non-ILD group. | | | | |
| --- | --- | --- | --- | --- |
|  | Total  (n=282) | PM/DM-ILD group  (n=140) | PM/DM-non-ILD group  (n=142) | *P* Value |
| **Clinical characteristics** | | | | |
| Age(y) | 53.4±13.2 | 56.1±11.8 | 49.0±13.6 | <0.001 |
| Gender (female), n (%) | 191 (67.7) | 101 (72.1) | 90 (63.4) | 0.185 |
| Hypertension, n (%) | 35 (12.4) | 22 (15.7) | 13 (9.2) | 0.208 |
| Diabetes mellitus, n (%) | 30 (12.1) | 11 (7.9) | 19 (13.4) | 0.709 |
| Smoking, n (%) | 70 (28.0) | 41 (29.3) | 29 (20.4) | 0.007 |
| Alcohol, n (%) | 44 (15.6) | 26 (18.6) | 18 (12.7) | 0.247 |
| Fever, n (%) | 65 (23.0) | 40 (28.6) | 25 (17.6) | 0.039 |
| **Laboratory indicators** | | | | |
| LDH, (U/L) | 299.0 (208.0-446.0) | 328.0 (229.0-471.0) | 223.0 (187.0-335.0) | <0.001 |
| CK, (U/L) | 175.0 (63.0-877.2) | 174.0 (56.0-1095.8) | 143.0 (70.0-542.7) | 0.507 |
| CRP, (mg/L) | 3.7 (1.2-15.2) | 5.1 (1.2-17.5) | 2.4 (0.9-9.1) | 0.032 |
| ESR, (mm/h) | 25.0 (9.0-45.5) | 27.5 (12.0-51.5) | 22.0 (5.0-36.0) | 0.012 |
| NLR | 4.8 (2.9-6.8) | 5.0 (3.1-6.9) | 4.0 (2.7-6.2) | 0.072 |
| **Autoantibody Information** | | | | |
| anti-MDA5 antibody | 70 (24.8) | 47 (33.6) | 23 (16.2) | 0.001 |
| anti-ARS antibody | 85 (30.1) | 46 (32.9) | 39 (27.5) | 0.164 |
| anti-Ro52 antibody | 93 (33.0) | 69 (49.3) | 24 (16.9) | <0.001 |
| **Notes:** PM/DM-ILD, Polymyositis and dermatomyositis associated with interstitial lung disease; LDH, lactate dehydrogenase; CK, creatine kinase; CRP, C-reactive protein; ESR, erythrocyte sedimentation rate; NLR, neutrophil-to-lymphocyte ratio; anti-MDA5 antibody, anti-melanoma differentiation-associated gene 5 antibodies; anti-Jo1 antibody, anti-histidyl-tRNA synthetase autoantibody. | | | | |

| **Table S2.** Univariate and Multivariate Cox Proportional Hazards Regression Analyses Comparing the Progression and Stable Groups in PM/DM-ILD Patients. | | | | |
| --- | --- | --- | --- | --- |
|  | ***Univariate Logistics Regression*** | | ***Multivariate Logistics Regression*** | |
|  | OR(95%CI) | *P* value | OR(95%CI) | *P* value |
| **Clinical characteristics** | | | | |
| Age | 1.04 (1.02-1.06) | <0.001 | 1.05 (1.02-1.07) | <0.001 |
| Gender (female) | 0.68 (0.41-1.19) | 0.186 |  |  |
| Hypertension | 1.71 (0.74-3.95) | 0.212 |  |  |
| Diabetes mellitus | 1.19 (0.47-3.01) | 0.710 |  |  |
| Smoking | 2.34 (1.26-4.37) | 0.007 | 2.57 (1.18-5.62) | 0.018 |
| Alcohol | 1.54 (0.74-3.24) | 0.250 |  |  |
| Fever | 1.99 (1.03-3.85) | 0.041 | - | - |
| **Laboratory indicators** | | | | |
| LDH | 1.00 (1.00-1.00) | 0.057 |  |  |
| CK | 1.00 (1.00-1.00) | 0.233 |  |  |
| CRP | 1.01 (0.99-1.02) | 0.422 |  |  |
| ESR | 1.02 (1.01-1.03) | 0.005 | - | - |
| NLR | 1.08 (1.00-1.16) | 0.038 | - | - |
| **Autoantibody Information** | | | | |
| anti-MDA5 antibody | 2.97 (1.50-5.89) | 0.002 | 3.55 (1.60-7.87) | 0.002 |
| anti-ARS antibody | 1.79 (0.74-4.35) | 0.197 |  |  |
| anti-Ro52 antibody | 6.38 (3.10-13.12) | <0.001 | 5.13 (2.32-11.33) | <0.001 |
| **Notes:** OR, Odds Ratio; 95% CI, 95% Confidence Interval; PM/DM-ILD, Polymyositis and dermatomyositis associated with interstitial lung disease; LDH, lactate dehydrogenase; CK, creatine kinase; CRP, C-reactive protein; ESR, erythrocyte sedimentation rate; NLR, neutrophil-to-lymphocyte ratio; anti-MDA5 antibody, anti-melanoma differentiation-associated gene 5 antibodies; anti-Jo1 antibody, anti-histidyl-tRNA synthetase autoantibody. | | | | |

| **Table S3**. Anti-ARS–stratified and sensitivity Cox specifications for acute/subacute ILD progression. | | | |
| --- | --- | --- | --- |
| Sensitivity Cox specification | Predictor | HR (95% CI) | *P* value |
| Specification 1 (OP only) | Imaging pattern (OP vs non-OP) | 1.32 (1.05-1.72) | 0.042 |
| Specification 2 (anti-ARS only) | Jo-1 positive | 0.74 (0.37–1.49) | 0.405 |
|  | Non–Jo-1 anti-ARS positive | 1.10 (0.49–2.46) | 0.813 |
| Specification 3 (OP + anti-ARS) | Imaging pattern (OP vs non-OP) | 1.31 (0.98–1.75) | 0.065 |
|  | Jo-1 positive | 0.75 (0.37–1.50) | 0.416 |
|  | Non–Jo-1 anti-ARS positive | 1.13 (0.50–2.54) | 0.765 |
| **Notes:** Cox proportional hazards regression was performed under three prespecified specifications: (Specification 1) OP pattern only, (Specification 2) anti-ARS status only, and (Specification 3) OP pattern and anti-ARS status jointly. Reference categories were non-OP for imaging pattern and ARS-negative for anti-ARS status. All models used the same follow-up definition for acute/subacute progression. P values were obtained from Wald tests. | | | |

| **Table S4**. Sensitivity analysis using time-window Cox models and a 30-day landmark approach. | | | | |
| --- | --- | --- | --- | --- |
| Predictor | Acutemprogression (≤30 days) Cox | | Subacute progression (31-90 days) Cox, landmark at 30 days | |
|  | HR (95% CI) | *P* value | HR (95% CI) | *P* value |
| Risk group: Intermediate vs Low | 3.54 (1.00-12.49) | 0.049 | 6.77 (2.24-20.50) | <0.001 |
| Risk group: High vs Low | 11.21 (3.13-40.09) | <0.001 | 11.81 (3.17-44.00) | <0.001 |
| Age | 1.01 (0.98-1.05) | 0.557 | 1.05 (1.01-1.09) | 0.011 |
| Fever | 2.07 (0.98-4.36) | 0.056 | 2.93 (1.36-6.32) | 0.006 |
| CK | 1.001 (0.999-1.002） | 0.307 | 1.001 (1.000-1.002) | 0.073 |
| **Notes:** Acute model: all participants were included; event defined as progression within 30 days; participants without progression were censored at 30 days. Subacute (landmark) model: participants with progression within 30 days were excluded; the risk set comprised patients event-free and under follow-up at day 30 (n = 112). Time was reset to 0 at day 30, and the event was progression occurring between days 31 and 90; patients without progression were censored at day 90. | | | | |

| **Table S5.** Incremental prognostic value of the HRCT score for predicting disease progression in patients with PM/DM-ILD. | | | |
| --- | --- | --- | --- |
|  | Clinical factors | Risk stratification | Integrated Clinical-Risk Stratification |
| Chi-square | 17.45 | 31.6 | 51.6 |
| C-index | 0.642 | 0.702 | 0.764 |
| P value | - | < 0.001 | < 0.001 |
| NRI | reference | 0.190 (0.159-0.444) | 0.470(0.257-0.621) |
| P value | - | 0.262 | 0.002 |
| IDI | reference | 0.117 (0.071-0.279) | 0.218 (0.093-0.347) |
| P value | - | 0.204 | 0.002 |
| **Notes:** 95% CI, 95% Confidence Interval; PM/DM-ILD, Polymyositis and dermatomyositis associated with interstitial lung disease. | | | |
